# Medial open wedge osteotomy yields comparable stability to lateral open wedge procedure on the distal femur

**DOI:** 10.1371/journal.pone.0310869

**Published:** 2024-09-25

**Authors:** Roman Frederik Karkosch, Michael Schwarze, Tomas Smith, Maximilian Petri, Marc-Frederic Pastor, Hauke Horstmann

**Affiliations:** 1 Orthopaedic Surgery Department, Hannover Medical School (MHH), Hannover, Germany; 2 Johanna Etienne Krankenhaus Neuss, Neuss, Germany; 3 Städtisches Klinikum Braunschweig gGmbH, Braunschweig, Germany; University of Vigo, SPAIN

## Abstract

**Background:**

Supracondylar osteotomies are a frequently and successfully used technique in the treatment of coronal plane deformities and unicompartmental osteoarthritis of the knee. While lateral open wedge techniques are common for valgus deformities, the data about medial open wedge techniques for varus deformities is sparse. The aim of this study was to compare the biomechanical properties of medial and lateral open wedge osteotomies using a locking Tomofix^®^ plate (DePuy Synthes, Oberdorf, Switzerland). Our hypothesis was that there would be no difference regarding biomechanical outcome parameters between these two groups.

**Methods:**

Medial and lateral open wedge osteotomies were performed in composite bone model as routine. Each experimental group contained 6 constructs. Standardized osteotomy gaps of ten millimeters were performed and Tomofix^®^ plates were fixed to third generation composite bones. The constructs were subsequently mounted into a servohydraulic testing machine. Axial and torsional loadings were applied as described in previous experimental studies. All specimens were subject to a load to failure mode with the mechanism of failure being noted.

**Findings:**

Both experimental groups showed comparable biomechanical properties under axial and torsional loadings. Mean high force axial stiffness was 3772 N/mm for lateral and 4185 N/mm for the medial construct. Significant differences were noted for torsional stiffness under low- (0 N) and mid-force (150 N) loadings (*P* = 0.002; *P* = 0.009), favoring the medial open wedge constructs.

**Interpretation:**

Medial open wedge osteotomy yields comparable biomechanical stability to the lateral open wedge procedure on the distal femur in a composite bone model.

## 1 Introduction

Coronal plane deformities of the knee affect joint loading and often lead to pain and the development of unicompartmental osteoarthritis in long term. While only 10–15% of patients present a valgus malalignment, the majority of patients are affected by a varus deformity [1, 2]. After failure of conservative treatment, several surgical techniques to correct the alignment have shown good results in reestablishing a physiological joint line and providing pain relief. With increasing possibilities of biological joint restoration and in order to avoid knee arthroplasty, osteotomies are an attractive solution especially in younger patients [3]. In valgus deformities, both medial closed wedge and lateral open wedge osteotomies of the femur have shown good success rates [4–8].

On the distal femur, lateral plate fixations oftentimes cause recalcitrant irritation of the iliotibial band requiring implant removal [9]. Therefore, medially placed plate fixations appear desirable to reduce the amount of revisions surgeries [10].

In varus deformities, tibial sided osteotomies are a well-established and frequently used procedure to reduce loadings on the medial aspect of the knee joint [11]. Of interest, Micicoi et al. have shown that up to 45% of patients with varus deformity at the knee joint have femoral sided or partially femoral sided malalignment [12]. Akamatsu et al. presented data that prove oblique joint line after high tibial osteotomy has inferior clinical results [13]. Therefore, femoral sided correction in varus deformity is highly underrated.

Yet, in case of a femoral sided malalignment limited procedures are available.

Qi-Fang et al presented a case series of 15 patients in which the performed medial open-wedge osteotomy of the distal femur with double plate fixation. It showed adequate bone healing and good functional outcome [14].

To our knowledge there is no present study on the correction of a femoral sided cause of varus using a medial open wedge procedure.. Moreover, there is no technical note by any company promoting their plate to be used for a medial open wedge procedure on the distal femur.

The aim of this study was to exclusively compare the biomechanical properties of medial and lateral open wedge osteotomies using a locking Tomofix^®^ plate, despite the contrary surgical indication. Our hypothesis was that there would be no difference regarding biomechanical outcome parameters between these two groups.

## 2 Methods

Being a biomechanical study using composite bones, no approval by the local ethics committee was required. A total of 12 composite femurs (Sawbones^®^ Europe, Malmö, Sweden) were used in this biomechanical examination.

### 2.1 Biomechanical testing

Biomechanical setups were chosen according to previously published studies by Brinkmann et al. who examined lateral open and medial closed wedge techniques of the femur [15–17].

The proximal and distal parts of the sawbones^®^ were embedded in casting resin (Rencast FC 53, Huntsman Advanced Materials, Basel, Switzerland), with additional socked screws providing rotational stability within the potting on the distal part.

Single plane open wedge osteotomies with medial and lateral plate osteosynthesis using an angle stable tomofix plate were performed according to standard surgical procedures [18]. The following implants were used:

- 4 hole Tomofix lateral distal femur plate (141 mm, Tomofix, DePuy Synthes, Oberdorf, Switzerland) for lateral open wedge procedure.
- 4 hole Tomofix medial distal femur plate (112 mm, Tomofix, DePuy Synthes, Oberdorf, Switzerland) for medial open wedge procedure.

**Fig 1:**
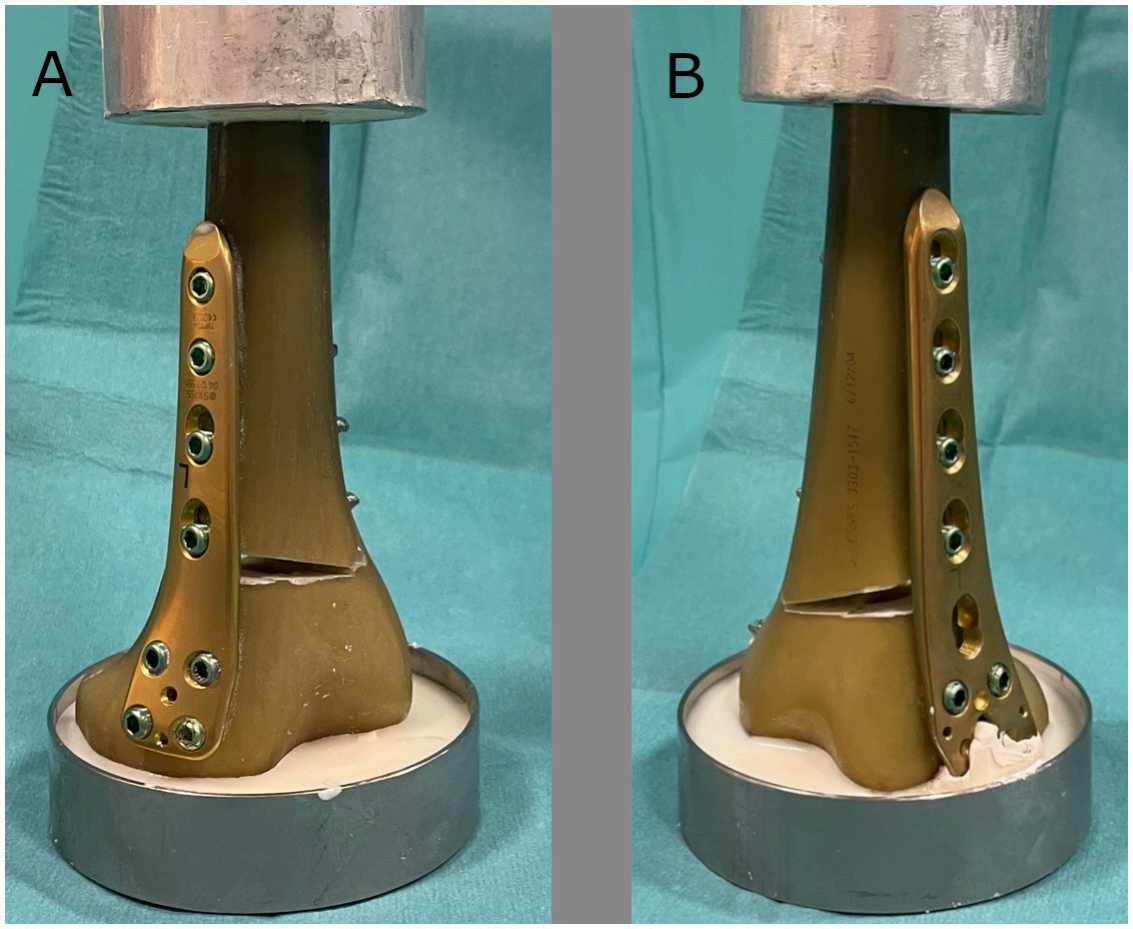
Embedded sawbone construct with tomofix plates. a) medial open wedge procedure; b) lateral open wedge procedure.

After precise positioning of the osteotomy gap all plates were fixed with eight angle stable locking screws to the composite bone to ensure a reproducible position. The gap size was chosen to be 10 mm in each case. This was ensured by implanting a triangular spacer which was removed after the plate was fixed to the femur. A bony cortex of 5mm length from an anterior perspective was left intact. In order to avoid a fracture of the remaining bone bridge the sawbone^®^ was slightly heated up during the process of implanting the spacer. This was achieved using a hot air fan. Fig 1 shows the embedded composite bone construct.

### 2.2 Testing protocol

Specimens were placed into a loading fixation of a servohydraulic testing machine (Mini Bionix 858; MTS Systems Co, Eden Prairie, USA). The proximal fixation was a universal joint in all cases (compare Fig 1). For axial and ultimate loading, the proximal part was placed in a ball and socket joint. For torsional loading, the proximal part was placed in a universal joint. For all tests, an initial preload of 10 N was applied.

All femurs were subject to an established loading protocol [19]. We applied 100 cycles of 50 to 150 N followed by 100 cycles of 50 to 800 N axial load at 1 Hz in a sinusoidal waveform. Torsional tests were performed with 100 cycles of 5 Nm internal rotational moment to 5 Nm external rotational moment at 0.25 Hz in a sinusoidal waveform under additional axial loads of 0 N, 150 N and 800 N, respectively.The described setup and values were chosen because of their resemblance of a postoperative partial weight bearing protocol following correction osteotomy starting with 15 kilograms and increasing to full weight bearing after four weeks post intervention.

Finally, all specimens were loaded to failure applying axial load at 0.1 mm/sec up to 8000 N. Premature failure was assessed macroscopically and defined as either fracture of the bony cortex or implant failure. A microscopical examination for possible damage to the osteotomy or implant was not performed.

Load and displacement of the actuator was continuously monitored by the testing machine at 1000 Hz.

### 2.3 Analysis

Axial stiffness was calculated for each loading cycle by dividing maximum force by maximum relative displacement. Torsional stiffness was calculated by dividing torque range by displacement range for each cycle. For the final analysis, all cycles of a loading step were averaged.

The Mann—Whitney-U-test for independent samples was used to compare stiffness of each loading mode and load-to-failure tests between plate setups. An alpha level of 0.05 was used as threshold for significance.

The sample size was determined by orientation on comparable biomechanical studies using composite bones, which exhibit lower variability of mechanical properties compared to human tissue.

## 3 Results

### 3.1 Main findings

No damages to the Sawbone^®^ constructs were evident throughout axial and torsional testing. No statistically significant difference could be noted between testing groups regarding most parameters.

Both experimental groups proved stable under axial and torsional loadings. Significant differences were noted for torsional stiffness under low and mid force loadings (*P* = 0.002; *P* = 0.009), favoring the medial open wedge constructs (Table 1).

**Table 1:** Overview of results for axial and torsional loadings for both testing groups.

	Axial stiffness (N/mm)		Torsional stiffness (Nm/deg)			Specimen
Load	150N	800N	0N	150N	800N	n
Lateral (mean)	2262 ± 964	3772 ± 918	5.29 ± 0.25	5.47 ± 0.29	6.56 ± 0.98	6
Medial (mean)	3474 ± 1357	4185 ± 822	6.36 ± 0.63	6.93 ± 0.83	7.76 ± 2.12	6
p-value	*0*.*093*	*0*.*70*	*0*.002*	*0*.009*	*0*.*24*	

### 3.2 Axial stiffness

**Fig 2:**
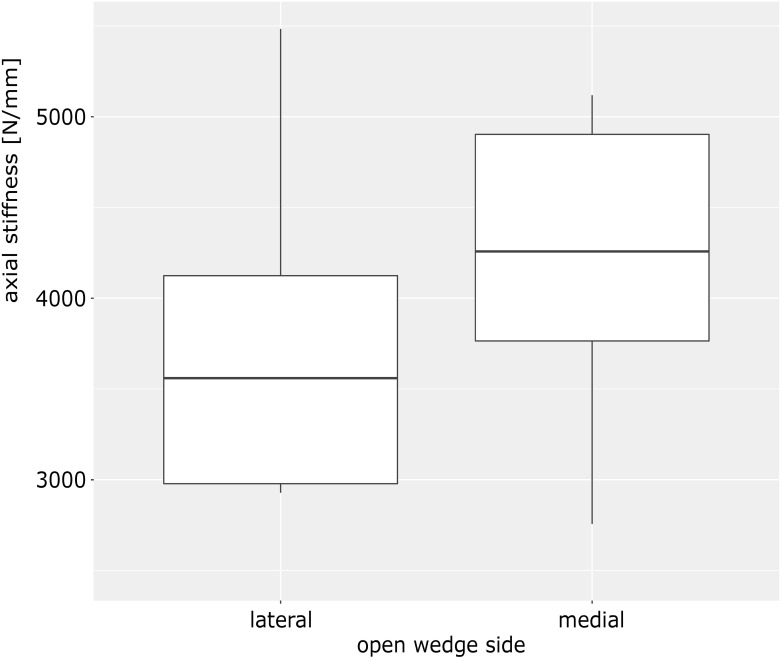
Axial stiffness under high force loading (800N).

No evident damage to the bone or implant was noted during cyclic loadings (Table 1). During axial loading protocol no statistically significant differences could be noted under low- and high force loadings (Fig 2). It should be noted that there is a tendency for an increased axial stiffness on the medial open wedge procedure.

### 3.3 Torsional stiffness

No evident damage to the bone or implant was noted during cyclic loadings (Table 1). Torsional stiffness was significantly greater in the medial open wedge group under low (0 N *P* = 0.002) and mid force (150 N; *P* = 0.009) loadings. No significant difference could be noted under high force loadings.

### 3.4 Failure mode

Failure mode for all failed specimens (lateral: n = 1, medial n = 2) was the breakage of the bony cortex. All other implants yielded the maximum load of 8000 N.

## 4 Discussion

The most important finding of the present study was that supracondylar medial open wedge osteotomies of the femur using Tomofix^®^ plate shows comparable biomechanical stability compared to the established lateral open wedge technique. As hypothesized, axial and torsional stiffness under high loadings as well as ultimate failure load did not show significant differences between testing groups.

While axial stiffness showed no significant differences under any loading, torsional stiffness was significantly greater in the medial open wedge group under low- (0 N *P* = 0.002) and mid-force (150 N; *P* = 0.009) loadings. Yet, it seems that negative effects of reduced torsional stiffness are less important to the constructs stability when they are in the range of what is required to grant a primary stability [15]. Biomechanically, higher stability in torsional as well as axial failure tests for close wedge compared to open wedge osteotomy has been proven [15] but this advantage in closed wedge osteotomies cannot be found in clinical trials [6–8].

In the present study, sawbones^®^ were used due to their previously described similarity to natural bones. These composite bones provide realistic biomechanical testing in a laboratory set up and have been used in many several other examinations in the past [15, 20]. Biomechanical key values like stiffness and longitudinal strain resemble the values of natural bones [20, 21]. Thus, the present trial provides a useful biomechanical preliminary work for clinical studies [16].

According to previous studies by Brinkmann et al. the test protocol was chosen to represent a postoperative regime of initial partial followed by full weight bearing [15–17].

Gap size and plate length are the decisive factors for postoperative stability in osteotomies [16, 22]. The gap size was consistent in our trial whereas the length of the plate differed between the medial (112 mm) and the lateral fixation (141 mm). Therefore the longer plate of the lateral open wedge osteotomy might be the more stable fixation. Surprisingly in our examination the shorter medial plate showed slightly increased values for stiffness under axial and torsional loadings. Nevertheless, the clinical influence of this advantage is questionable due to ongoing bone healing [16].

In previously published biomechanical exanimations the predominant failure modes were pertrochantic and femoral neck fracture [15]. Due to our setup this could be avoided as only the central third of the femur was embedded.

Advantages of open wedge osteotomy include the ability to reliably control the wedge size [23, 24] and a low risk of hinge fracture [25]. At the distal femur, laterally sided osteotomies with plate osteosynthesis bear the inherent high rate of postoperative implant removal due to irritation of the iliotibial band [9]. Therefore, medially placed plate fixations appear desirable to reduce the amount of revisions surgeries [10].

### 4.1 Limitations

Several limitations apply to this study. First, being a biomechanical study, the biological healing process and postoperative rehabilitation, which are crucial to the surgery’s outcome, could not be taken into account.

Second, we can only provide the time-zero data of biomechanical stability following osteotomy and locking plate osteosynthesis. In addition, due to the use of composite bones there is no biological or anatomical variability to be found among the constructs.

Moreover, in this examination composite bones instead of human femurs were used. Even though their stiffness values are supposed to resemble the ones of natural human bones, our experiment required slight preheating of the construct in order to be able to create an osteotomy gap of ten millimeters without producing an immediate hinge fracture. Therefore, we see a need for further in-vitro examinations, ideally using full-length human femurs to confirm the data acquired from this composite bone study. This is emphasized by the fact that to our knowledge there are no other studies examining a medial open wedge procedure on the distal femur.

## 5 Conclusion

Medial open wedge osteotomy yields comparable and in low and mid force torsional stiffness superior biomechanical stability compared to the lateral open wedge procedure on the distal femur in a composite bone model.

## Supporting information

S1 File Raw data of all specimens tested. This file contains all raw data in csv format as it was stored on the testing machine.
(ZIP)
